# North Atlantic Heat Transport Convergence Derived from a Regional Energy Budget Using Different Ocean Heat Content Estimates

**DOI:** 10.1007/s10712-024-09865-5

**Published:** 2024-10-24

**Authors:** B. Meyssignac, S. Fourest, Michael Mayer, G. C. Johnson, F. M. Calafat, M. Ablain, T. Boyer, L. Cheng, D. Desbruyères, G. Forget, D. Giglio, M. Kuusela, R. Locarnini, J. M. Lyman, W. Llovel, A. Mishonov, J. Reagan, V. Rousseau, J. Benveniste

**Affiliations:** 1 Université de Toulouse, LEGOS (CNES/CNRS/IRD/UT3), 31400 Toulouse, France; 2 Research Department, European Centre for Medium-Range Weather Forecasts, 53175 Bonn, Germany; 3 Department for Meteorology and Geophysics, University of Vienna, 1090 Vienna, Austria; 4 NOAA/Pacific Marine Environmental Laboratory, Seattle, Washington 98115 USA; 5 National Oceanography Centre, Liverpool, L3 5DA UK; 6 MAGELLIUM, 31250 Ramonville Saint-Agne, France; 7 NOAA National Centers for Environmental Information, Silver Spring, MD 20910 USA; 8 Institute of Atmospheric Physics, Chinese Academy of Sciences, Beijing, 100029 China; 9 CNRS, Ifremer, IRD, Laboratoire d’Océanographie Physique et Spatiale (LOPS), IUEM, University of Brest, 29280 Plouzané, France; 10 Department of Earth, Atmospheric and Planetary Sciences, Massachusetts Institute of Technology, Cambridge, MA 02139-4307 USA; 11 Department of Atmospheric and Oceanic Sciences, University of Colorado Boulder, Boulder, CO 80309-0311 USA; 12 Department of Statistics and Data Science, Carnegie Mellon University, Pittsburgh, PA 15213 USA; 13 CIMAR, University of Hawaii, Honolulu, HI 96822 USA; 14 Cooperative Institute for Satellite Earth Systems Studies, Earth System Science Interdisciplinary Center, University of Maryland, College Park, MD 20742 USA; 15 European Space Agency (ESA-ESRIN), 00044 Frascati, Italy

**Keywords:** North Atlantic heat transport, Regional energy budget, Energy transport, Climate variability, Energy budget/balance, Heat budgets/fluxes, Surface fluxes, In situ observations, Satellite observations, Ocean heat content

## Abstract

This study uses an oceanic energy budget to estimate the ocean heat transport convergence in the North Atlantic during 2005–2018. The horizontal convergence of the ocean heat transport is estimated using ocean heat content tendency primarily derived from satellite altimetry combined with space gravimetry. The net surface energy fluxes are inferred from mass-corrected divergence of atmospheric energy transport and tendency of the ECMWF ERA5 reanalysis combined with top-of-the-atmosphere radiative fluxes from the clouds and the Earth’s radiant energy system project. The indirectly estimated horizontal convergence of the ocean heat transport is integrated between the rapid climate change-meridional overturning circulation and heatflux array (RAPID) section at 26.5°N (operating since 2004) and the overturning in the subpolar north atlantic program (OSNAP) section, situated at 53°–60°N (operating since 2014). This is to validate the ocean heat transport convergence estimate against an independent estimate derived from RAPID and OSNAP in-situ measurements. The mean ocean energy budget of the North Atlantic is closed to within ± 0.25 PW between RAPID and OSNAP sections. The mean oceanic heat transport convergence between these sections is 0.58 ± 0.25 PW, which agrees well with observed section transports. Interannual variability of the inferred oceanic heat transport convergence is also in reasonable agreement with the interannual variability observed at RAPID and OSNAP, with a correlation of 0.54 between annual time series. The correlation increases to 0.67 for biannual time series. Other estimates of the ocean energy budget based on ocean heat content tendency derived from various methods give similar results. Despite a large spread, the correlation is always significant meaning the results are robust against the method to estimate the ocean heat content tendency.

**Article Highlights**

- The mean ocean energy budget of the North Atlantic is closed, within ± 0.25 PW, between RAPID and OSNAP sections
- The inferred mean oceanic heat transport convergence between RAPID and OSNAP sections is 0.58 ± 0.26 PW, which agrees well with observed section transports
- Interannual variability of the North Atlantic oceanic heat transport convergence is in reasonable agreement with the interannual variability observed at RAPID and OSNAP, with a correlation of 0.54 between annual time series and 0.67 for biannual time series
- The results are robust against those products used to estimate the ocean heat content (OHC) tendency, whether these products are based on in situ measurements, satellite altimetry, and space gravimetry or a combination of them

## Introduction

The Atlantic meridional overturning circulation (AMOC) is often represented as stream function in latitude-depth or sometimes latitude-density space derived from the zonally-integrated meridional velocities in the Atlantic Ocean (Frajka-Williams et al. 2019; Jackson et al. 2019; Rousselet et al. 2021). It is characterized by a northward flow of warm, salty upper ocean water, and a southward flow of colder, denser, deep waters that sink in the subpolar North Atlantic and Greenland-Iceland-Norwegian Sea after transferring heat to the atmosphere. The AMOC affects a meridional transport of heat (MHT) in the North Atlantic basin that is positive northward. It is responsible for most of the meridional transport of heat by the midlatitude northern hemisphere ocean (and up to 25% of the northward global atmosphere–ocean heat transport in the northern hemisphere -e.g., Bryden et al. 2001-). This North Atlantic MHT plays a crucial role in the climate variability of the entire Northern hemisphere, particularly affecting ocean temperature and circulation, and the Atlantic storm track (e.g., Rhines et al. 2008). Additionally, the Atlantic MHT impacts the regional climate by warming the atmosphere over Europe, thereby influencing air temperature and precipitation in this densely populated region (e.g., Palter 2015). Future projections with global warming suggest a weakening of the North Atlantic MHT with potential profound local climate effects. However, there are substantial disagreements among models both in terms of how well they simulate the MHT and the magnitude of future changes (e.g. Collins et al. 2019; Mecking and Drijfhout 2023). Accordingly, there is considerable interest in quantifying the North Atlantic variability to better validate and constrain climate simulations and determine whether the projected trends are a response to the forced climate or internal variability.

**Fig. 1:**
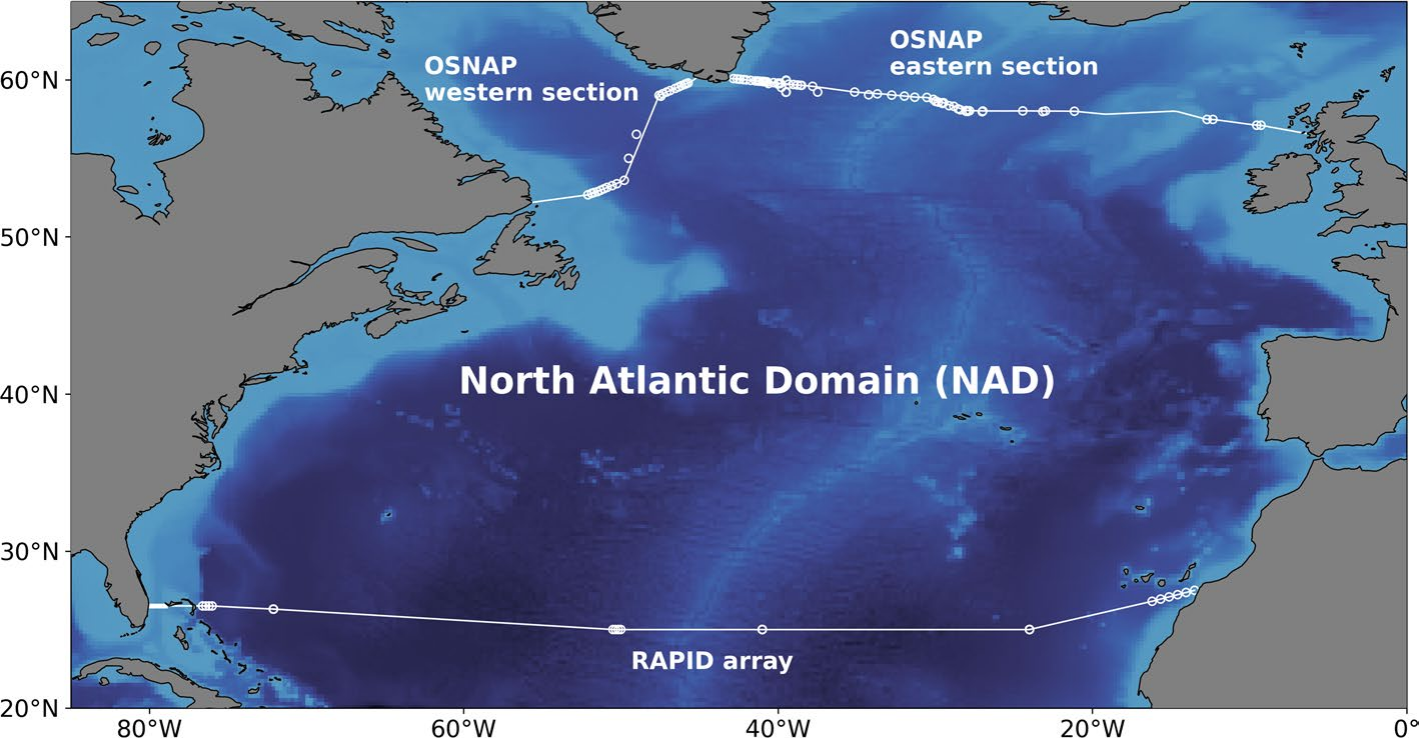
Map of the North Atlantic domain (NAD) in which the ocean energy budget is evaluated. The southern white line indicates the 26.5°N latitude where the RAPID array is located. The white dots along the line indicate the position of the RAPID array moorings. The Northern white lines indicate the two legs of OSNAP and the dots indicate the location of the OSNAP moorings

In this work, we evaluate the zonal mean MHT in the North Atlantic as a residual of the ocean energy budget (see Sect. 2). An advantage of this approach is that it provides estimates of the zonal mean MHT at any latitude. For validation, we compare our estimate of the zonal mean MHT with estimates derived from in situ measurements of the RAPID array, at the RAPID section at 26.5°N, and of the OSNAP array, along OSNAP sections in the subpolar North Atlantic (see Fig. 1 and Sect. 4). We further analyze the spatio-temporal variability of the North Atlantic MHT and its causes (see Sect. 4). As the MHT is derived from an energy budget, we evaluate the role of the different terms of the oceanic energy budget in the North Atlantic MHT spatiotemporal variability (see Sect. 4 and 5).

The indirect method that we use here to evaluate the zonal mean MHT in the North Atlantic (which relies on an ocean energy budget) has already been developed in the literature (Mayer et al. 2022; Liu et al. 2020, Trenberth and Fasullo 2017). Previous studies use atmospheric reanalysis (ERA-interim -Dee et al. 2011 and ERA5—Hersbach et al. 2020) combined with top-of-the-atmosphere (TOA) radiative fluxes (from the clouds and the earth’s radiant energy system project-CERES) to estimate the net surface fluxes and ocean reanalyses (ORAS5 -Zuo et al. 2019 and ORAP6 -Zuo et al. 2021) to estimate the ocean heat content tendency (OHCT). They show that the ocean heat content tendency term of the energy budget explains most of the time variability in the Atlantic MHT at the RAPID section on interannual and longer time scales. However, it has never been explored the extent to which the estimate of the Atlantic MHT time variability is dependent on the North Atlantic OHCT estimate derived from ocean reanalysis.

In this study, we use a recent estimate of the ocean heat content changes over the North Atlantic derived from a geodetic approach which combines satellite altimetry data with space gravimetry data (update of Marti et al. 2022, 2024) to estimate the OHCT. We also use other estimates of the OHCT either derived from in situ measurements of the ocean temperature by the Argo network of profiling floats augmented by other ocean observing systems, or derived from in situ data combined with satellite altimetry data, or derived from an ocean reanalysis. With this ensemble of OHCT estimates, we assess the closure of the ocean energy budget and evaluate the spread in MHT that is induced by the differences across different OHCT estimates. This approach has three important benefits: First, it enables exploration of the sensitivity of the North Atlantic MHT estimate on the OHC product that is used and thus provides insights on the uncertainty in the MHT variability that is due to the uncertainty in the OHC estimate (which is likely the largest source of uncertainty in the estimated MHT variability). Second, it enables testing for closure of the North Atlantic Ocean energy budget. This important piece of information reveals whether the important causes of the North Atlantic MHT are identified and how closely their combination matches the MHT estimated using mooring data at RAPID and OSNAP sections. It provides a quantitative understanding of why and how the North Atlantic MHT is changing with time. It also cross-validates different products based on different observations such as the OHCT based on satellite altimetry and space gravimetry or argo observations, the atmospheric lateral energy transport divergence and tendency of ERA5, and the TOA radiative fluxes from CERES. This cross-validation is an efficient approach to evaluate the consistency of different observed North Atlantic variables including OHCT, TOA radiation fluxes, and MHT with regard to the energy conservation law. This aspect is particularly important when we want to use OHCT products, TOA radiation products, and MHT products in globally consistent objective estimates of the global energy budget such as the one developed in the world climate research program global energy and water exchanges (GEWEX) project (Stephens et al. 2023) and in the NASA NEWS project (e.g., L’Ecuyer et al. 2015 and Roberts et al. this issue). We evaluate and discuss these three benefits in the discussion section.

## Method

The oceanic energy budget is evaluated over a closed domain of the North Atlantic region, between the RAPID section and the OSNAP sections, for the period of 2005–2018. The OSNAP sections comprise two legs: one leg from Southern Labrador to South-west Greenland and one leg from South-east Greenland to the coast of Scotland (see Fig. 1). The Mediterranean sea is excluded from the domain. This closed North Atlantic domain is called NAD hereafter.

We write the vertical integral of the oceanic energy budget for any column of water located in NAD as

1 $$\nabla .{\text{OHF}} = {\text{SHF}} - {\text{OHCT}}$$

where $\nabla .{\text{OHF}}$ is the vertical integral of the horizontal divergence (i.e., the opposite of the horizontal convergence) of the ocean heat transport in the water column, ${\text{SHF}}$ is the net surface heat flux at the top of the water column, and ${\text{OHCT}}$ is the temporal ocean heat content tendency of the water column. Since 2000s, Sea ice is only presented in the NAD in a narrow band along southern Labrador in winter, so the tendency in the sea ice melt energy is neglected in Eq. (1).

When $\nabla {\text{OHF}}$ is integrated horizontally over any large domain such NAD, it corresponds to the heat transport convergence or to the difference in MHT through the frontiers of the domain (Gauss theorem). In the case of the NAD, the horizontal integration of $\nabla {\text{OHF}}$ represents the difference between the MHT through the RAPID section and the MHT through the OSNAP sections.

In this study, the vertical integral of the convergence of the ocean heat transport (i.e., the integral of $- \nabla {\text{OHF}}$) is estimated as a residual of Eq. (1). The surface heat flux is derived as the residual of the mass-corrected atmospheric energy budget as in Mayer et al. (2017) and discussed also in this issue (Mayer et al. 2024). That is, the surface heat flux is estimated as the difference of the TOA net radiative fluxes with the atmospheric tendency of energy and the vertically integrated divergence of atmospheric moist static plus kinetic energy fluxes. The OHCT is estimated by derivation of the ocean heat content which is derived either from optimal interpolation of in situ measurements of the ocean temperature and salinity (with a vertical integration of the specific heat of sea water multiplied by the local density of seawater and the oceanic temperature as in Melet and Meyssignac 2015), or from the observed thermal expansion of the sea water (with the local thermosteric sea level rise multiplied by the local integrated expansion efficiency of heat, e.g., Marti et al. 2022), or from the reconstruction of the ocean state by an ocean reanalysis.

Small enthalpy fluxes, associated to evaporation, precipitation, river run-off, and the inflow from the Mediterranean, are entering or leaving the NAD. In total, these enthalpy fluxes are of the order of 10TW (the dominating enthalpy flux is due to the Mediterranean inflow and amounts less than 15TW on average over decadal time scales, Macdonald et al. 1994; Wu and Haines 1998). 10TW is about two orders of magnitude smaller than the different terms of the budget represented in Eq. (1) so we neglect these enthalpy fluxes in the ocean energy budget. Small amounts of mass may enter or leave the NAD without going through the RAPID and OSNAP sections. It can be sea ice floating in and out through the northern boundary, evaporation and precipitation through the surface, river run-off and river discharge through the coast, or the inflow from the Mediterranean. It is also likely that RAPID and OSNAP do not fully sample all the net mass flux going through their respective sections. This small amount of mass generates a bias in the NAD oceanic energy budget (see Mayer et al. 2022 for more details). A mass correction should be applied to the ocean energy budget to balance RAPID, OSNAP, and other term volume fluxes. However, in this study, we use heat transports at both RAPID and OSNAP which are computed with a flow field that is constrained to have zero net mass transport through both sections. This constraint on the flow field implicitly accounts for precipitation minus evaporation plus other small input of water in the NAD region. So, the mass correction is not needed here.

## Data

### Data Used to Estimate the Net Surface Heat Flux ${\text{SHF}}$

The net surface heat flux ${\text{SHF}}$ is inferred over the period of 2005–2018 from the mass corrected vertically integrated total atmospheric energy budget in which, the mass-balanced atmospheric horizontal energy transport divergence and the atmospheric energy tendency are derived from ECMWF’s latest reanalysis dataset ERA5 as in Mayer et al. (2021a). ERA5 provides a four-dimensional estimate of the atmospheric state at ~ 31 km spatial and hourly temporal resolution, generated using a 4-dimensional variational data assimilation method that ingests a wealth of remotely sensed and in situ-based observational information (Hersbach et al. 2020). The atmospheric budget data are available from the Copernicus Climate Data Store (CDS; Mayer et al. 2021b). The net TOA radiation fluxes are derived from the CERES–Energy Balanced and Filled (CERES-EBAF) product in version 4.1 (Loeb et al. 2018*).* The uncertainty on the net surface heat flux is evaluated using two other atmospheric reanalysis (namely JRA55 and MERRA2) to estimate the net surface heat flux ${\text{SHF}}$. For each month, we consider the maximum difference across the three reanalysis estimates of the SHF as the standard deviation of the uncertainty for this monthly SHF estimate. Note that we could have considered the ensemble mean of the three reanalysis estimates of the SHF as the best estimate of the SHF, but the literature suggests that the ERA5 reanalysis performs best for ocean energy budgets (see Mayer et al. 2022) so we use ERA5 estimate as best estimate.

### Data Used to Estimate the Ocean Heat Content Tendency ${\text{OHCT}}$

${\text{OHCT}}$ is estimated from eight different products in total, comprising ocean in situ temperature products from the National Oceanographic and Atmospheric Administration (NCEI from Levitus et al. 2012), from Ifremer (ISAS21 Kolodziejczyk et al. 2023), from the National Oceanic Center (NOC, King 2023), by Giglio et al. 2023 (LocalGPspace), and from the Institute of Atmospheric Physics (IAP, Cheng et al. 2017), plus Ocean heat content products from the European Space Agency (Magellium/LEGOS, 2022; Marti et al. 2024 in revision), NOAA/PMEL (RFROM, Lyman and Johnson 2023), and an ocean state reanalysis (ECCO, Fukumori et al 2021). The ECCO and Magellium/LEGOS estimates are full depth, but the others are from the surface to 2000 m depth.

The ocean in situ products consist of statistical optimal interpolation of the observed in situ temperature profiles mainly from the argo profiling float network over the period of 2005–2018. The argo data are augmented by ocean temperature observations from ship-based observations (research ships and ship of opportunity merchant ships), moored buoys (mainly the tropical moored buoy array with additional OceanSITES buoys) and sometimes gliders, and pinniped mounted sensors.

The NCEI product is a gridded product generated by objective analysis of binned one-degree latitude/longitude means of monthly temperature anomalies calculated from all available ocean profile temperature data subtracted from the corresponding one-degree climatological mean temperature at standard depth levels for years of 1955–2006 (World Ocean Atlas 2009, Locarnini et al. 2010) as described in Levitus et al. 2012. Analyzed temperature anomalies are added back to the climatological mean field to obtain temperature and salinity fields for the time period in question (each month for years 2005–2022) at 26 vertical levels between the surface and 2000 m.

The ISAS21 product is a gridded product of temperature and salinity data derived from the in situ analysis system (ISAS, Gaillard et al. 2016). This version is an update of ISAS21_ARGO (https://www.seanoe.org/data/00412/52367/data/86436.pdf). This product merges all the available delayed-mode quality-controlled (core and deep) argo profiles in the Atlantic domain along with oceanographic campaigns. ISAS uses an optimal interpolation scheme that preserves as much as possible the time and space sampling capabilities of the in situ profiles. The ISAS procedure and products are described in Gaillard et al. (2016).and in Kolodziejczyk et al. (2023).

The NOC_OI product provides gridded fields of in situ temperature and practical salinity encompassing the period from 2004 to 2022. Such fields have been generated through objective mapping of argo profiling float data and quality-controlled mooring data at 26.5^°^N from RAPID. The data are provided on a 1° × 1° grid with a vertical spacing of 20 decibars and a temporal resolution of 10 days. Quality control procedures were conducted by the source data programs, and no additional quality control was applied before the mapping process.

The LocalGPspace product (Giglio et al. 2023) provides OHC fields that are mapped using locally stationary Gaussian processes with data-driven decorrelation scales (Kuusela and Stein 2018). A linear time trend is included in the estimate of the mean field, along with spatial terms and harmonics for the annual cycle. Mapping is done separately for different vertical sections, which are then combined to estimate global OHC timeseries in the upper 2000 dbar of the ocean. Regions of the ocean that are not sufficiently well sampled by the Argo array are not included.

The IAP product is a gridded product generated by the Institute of Atmospheric Physics (IAP hereinafter, Cheng and Zhu 2016; Cheng et al. 2017). It has advantages in both instrumental error reduction and its gap-filling method. IAP mapping technique used spatial covariance from model simulations to help provide spatial interpolation. This product merges all the available bias-corrected in situ ocean temperature observations from a variety of instruments held in the World Ocean Database. The spatial resolution of IAP data is 1° by 1° mesh grid from 1 to 2000 m (41 levels), and the temporal resolution is monthly.

The ocean heat content products consist of a combination of ocean in situ temperature profiles with satellite data derived from satellite altimetry and space gravimetry.

The Magellium/LEGOS product is a gridded OHC product available from April 2002 to December 2020 over the Atlantic Ocean (Magellium/LEGOS, 2022). The spatial resolution is 1° in latitude and longitude with a monthly temporal resolution. The product is the combination of satellite altimetry-based data (C3S, Legeais et al. 2021) and satellite gravimetry-based data (update of Blazquez et al. 2018) to estimate local expansion of sea water in addition to in situ data (EN4.2.2.l09, Levitus et al. 2009, and ISAS21, Kolodziejczyk et al. 2021) to correct for the salinity effect and convert the resulting thermal expansion into OHC with the integrated expansion efficiency of heat. Uncertainties are provided regionally at a yearly timescale in a variance–covariance matrix and are estimated by propagating uncertainties from the input satellite data until the OHC change. The regional uncertainties associated with altimetry data are derived from the error budget of Prandi et al. (2021) while uncertainties associated with gravimetry data are estimated with an ensemble approach derived from Blazquez et al. (2018). The Magellium/LEGOS product and its uncertainty are described in Marti et al. (2024) and references therein. From the local variance–covariance matrix of the uncertainties of the Magellium/LEGOS product, we compute the variance of the uncertainty in OHCT at each time step for each location in the NAD. The uncertainty variance–covariance matrix of the Magellium/LEGOS product only includes the temporal correlation of the errors and does not include the spatial correlation in errors. We adopt a conservative approach and consider that the Magellium/LEGOS product errors are fully correlated spatially. So, we add linearly the uncertainties of the OHCT of each location when the OHCT is aggregated over the NAD to estimate the uncertainty on the OHCT spatial mean. Among all OHC products, the LEGOS/Magellium product is the most comprehensive one as it covers the global ocean down to the bottom of the ocean and it provides an uncertainty estimates which accounts for all sources of known errors including the time correlation in errors. For this reason, we use this product to test wether the NAD energy budget (on Fig. 5) is closed within uncertainties.

RFROM (random forest regression ocean maps) is a gridded product of ocean heat content anomaly maps produced with a machine learning algorithm, random forest regression. In situ ocean temperature profile data are used as training data. Geographic location, time, and gridded satellite sea-surface height (SSH) and sea-surface temperature (SST) maps are used as predictors. The end result is ocean heat content anomaly maps for 10 different pressure levels between the ocean surface and 2000 dbar at 7-day × ¼° × ¼° resolution, starting in January 1993 (Lyman and Johnson, 2023).

The ocean reanalysis is the ECCO4 reanalysis which estimates the ocean state from 1992 to 2017 from a collection of global datasets (Forget et al 2015a; Fukumori et al 2021). ECCO4 notably includes argo, altimetry, gravimetry, and atmospheric data as constraints. The fit of model to data is achieved through optimization of forcing fields and parameters that control turbulent transport rates in the ocean interior (Forget et al 2015a, b; Forget and Ponte 2015). Through this technique, ECCO4 provides a dynamically consistent estimate of ocean transports, OHU, and OHC variability that form a closed heat budget unlike other data assimilative products that involve state variable increments of unknown nature (Storto et al 2019). A detailed analysis of ECCO4’s heat budget in terms of meridional transports is available in Forget and Ferreira 2019.

### Data Used to Validate the Vertical Integral of the Oceanic Energy Budget $\nabla {\text{OHF }}$

The vertical integral of the horizontal divergence of the ocean heat transport $\nabla {\text{OHF}}$ is inferred from the net surface heat flux $\left( {{\text{SHF}}} \right)$ and the ocean heat content tendency $\left( {{\text{OHCT}}} \right)$ (following the equation of the oceanic energy budget, Eq. (1)). Then, it is integrated horizontally over the NAD and compared with the difference of the MHT between the RAPID section and the OSNAP sections for validation.

The MHT across the RAPID (26.5^°^N) and OSNAP (between 50 and 60^o^N) trans-basin sections is calculated by integrating the product of the cross-sectional velocity, potential temperature, specific heat and density along each section based on data from mooring arrays, argo floats, and (at OSNAP) gliders. The MHT due to Ekman transport is derived from the wind fields of the ERA5 reanalysis (RAPID) and the ERA-Interim reanalysis (OSNAP). At the RAPID section, MHT through the Florida Straits is estimated based on measurements from a submarine cable. For RAPID, the data are available as 12-hourly estimates of MHT spanning the period from April 2004 to August 2018, whereas for OSNAP, the data are provided as 30-day mean estimates for the period from August 2014 to May 2018. We note that these transports represent true heat transports, at both RAPID and OSNAP, since the flow field used in the calculation is constrained to have zero net mass transport through both sections. The approaches to calculate MHT at the RAPID and OSNAP sections are described, respectively, by Johns et al. (2011) and Lozier et al. (2019), and we defer to those studies for full details. For the MHT uncertainties at the RAPID section, we consider an uncertainty of ± 0.22 PW (at 1 sigma) on the monthly time series that is derived from the ± 0.21 PW uncertainty on daily times series from Johns et al. (2011) onto which we added a possible measurement bias error estimated to be  ± 0.07 PW (W. Johns personal communication). Since only one degree of freedom per 40 days of observations were considered in the Johns et al. (2011) uncertainty estimate, the error bar on the RAPID MHT monthly time series is estimated to be same as the daily error bar. For the MHT uncertainties at the OSNAP sections, we use the uncertainties given in the product for the period of 2015–2018. For the period of 2005–2014, we use the same uncertainty at each month calculated as the mean monthly uncertainty over the period of 2015–2018 (i.e., ± 0.1 PW at 1 sigma). We consider the uncertainty in the OSNAP MHT and the RAPID MHT as uncorrelated because they are derived from independent data. So, we add quadratically their uncertainty to estimate the uncertainty on the NAD heat transport convergence.

All calculations in this work are done with monthly time series of the different datasets on their native grid. The datasets are then resampled on the same 1° × 1° grid at the last step and then combined together to infer the vertical integral of the oceanic energy budget. The climatology is then removed to get anomalies. When time series are filtered in time, we use the same Lanczos filter to filter them all in a consistent way. All correlations are Pearson correlations and the associated confidence intervals is estimated with a two-tailed p-value test.

## Results

### The Mean Ocean Energy Budget over 2005–2018

**Fig. 2:**
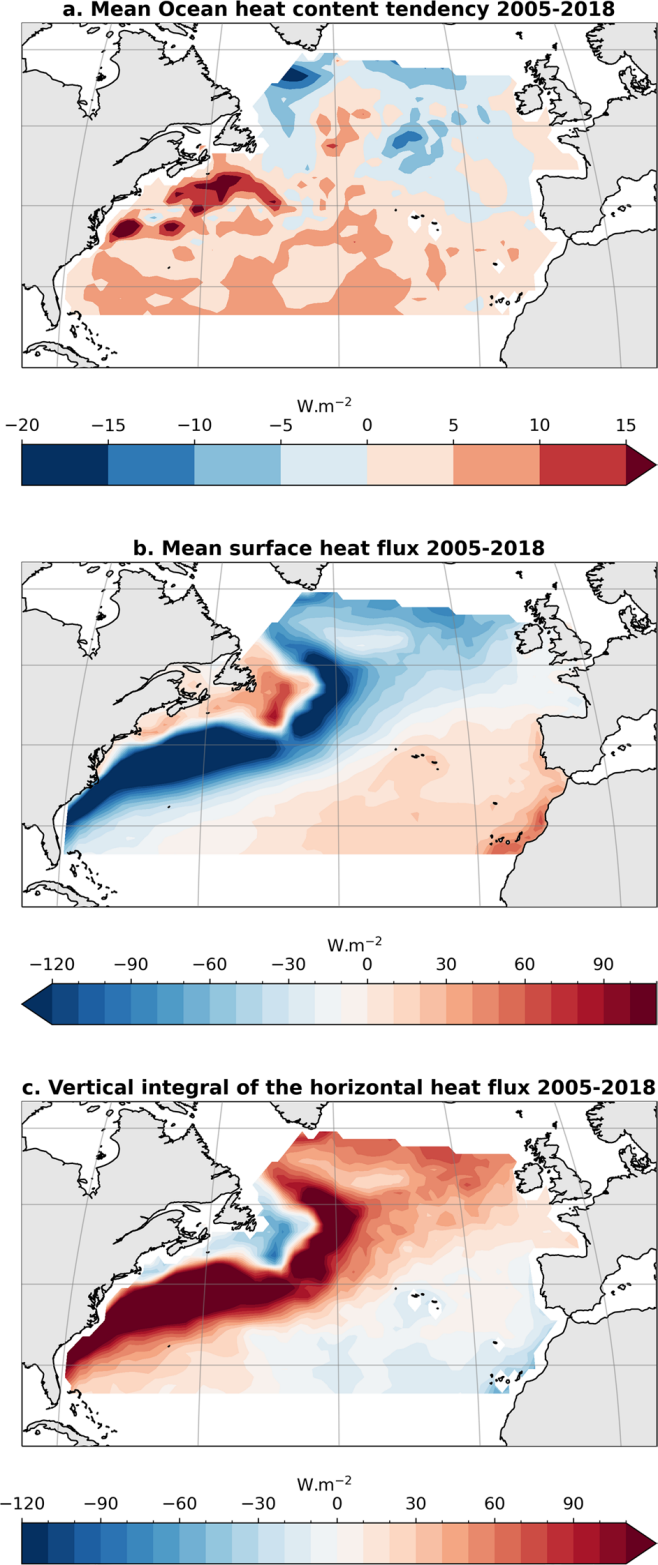
Maps of North Atlantic mean ocean heat content tendency (mean OHCT) (**a**), mean surface heat flux derived from ERA5 and CERES (mean SHF) (**b**), and mean vertical integral of the horizontal heat flux convergence inferred from the energy budget residual (mean $\nabla {\text{OHF}}$) (**c**), for the period of 2005–2018. In **a** and **c**, the OHCT is derived with the geodetic method from the Magellium/LEGOS product (see Fig. 3 For the OHCT over the same period derived from other OHC products)

The mean surface heat flux is the dominant term of the mean NAD energy budget from 2005 to 2018 in both amplitude and spatial variability (Fig. 2b). Over 2005–2018, the surface heat flux shows a mean heat loss from the ocean to the atmosphere of 0.54 PW over the NAD. Ocean heat uptake in the tropics is discharged from the ocean to the atmosphere at higher latitudes and in particular along the Gulf stream (Fig. 2b). This picture is consistent with the general poleward redistribution of heat in the upper ocean. This air-sea heat flux is about three times as large as the mean OHCT in the region (Fig. 2a).

**Fig. 3:**
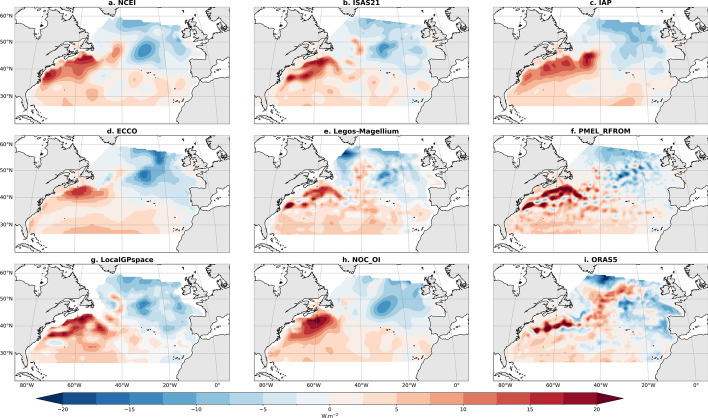
OHCT averaged over 2005–2018 derived from the different OHC products considered in this study. The middle panel corresponds to Fig. 2 panel a

Over 2005–2018, the time-mean OHCT is small on average over the NAD (0.062 PW), with warming generally in the subtropics that is strongest in the north portions of the Gulf stream and cooling through much of the subpolar region (Fig. 2a). This spatial variability is consistent across all estimates of the mean OHCT whether they are based on satellite data, in situ observations, or reanalysis (Fig. 3). The OHCT estimates using satellite altimetry data tend to show finer spatial structures coming from the mesoscale activity partially resolved by satellite radar altimeters, in particular along the Gulf stream and North Atlantic current (Fig. 3a,b). When combined together, the different terms of the mean ocean energy budget lead to an estimate of the time-mean vertical integral of the horizontal heat flux that shows a maximum along the Gulf stream and at high latitude (Fig. 2d). This pattern is owing to the role of the mean surface heat flux, which dominates the mean energy budget. The spatial variability of the OHCT is small and the spatial variability of the mean vertical integral of the horizontal heat flux convergence is actually almost all compensated for by the spatial variability of the mean surface heat flux.

### Meridional Heat Transport Between the RAPID and OSNAP Sections over 2005–2018

**Fig. 4:**
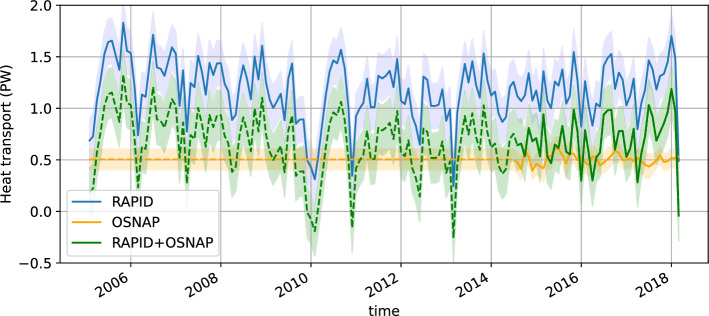
OSNAP (yellow plain line) and RAPID (blue line) meridional heat transport estimated from, respectively, OSNAP and RAPID arrays in situ observations. Approximation of OSNAP meridional heat transport with a constant of 0.5 ± 0.05 PW over 2005–2014 (yellow dashed line). NAD heat transport convergence from the difference of RAPID and OSNAP meridional heat transport (green line)

Poleward heat transport of 0.50 ± 0.1 PW is estimated to cross the OSNAP sections from August 2014 to May 2018 (Fig. 4). The overturning circulation is largely responsible for setting this heat transport (Li et al. 2021). In contrast, the RAPID array indicates a much larger mean heat transport across 26°N of 1.18 ± 0.22 PW over August 2014 to May 2018 and of 1.17 ± 0.22 PW over 2005–2018 (Fig. 4b). These results are expected as the Atlantic poleward heat transport is known to be stronger in the subtropical North Atlantic than in the subpolar North Atlantic (Ganachaud and Wunsch 2000, Trenberth et al., 2001). At the RAPID section, the overturning circulation is also largely responsible for the MHT, even more than at OSNAP sections (McCarthy et al., 2015). The comparison of the RAPID and OSNAP MHT estimates shows another difference: the subtropical MHT is markedly more variable over monthly to interannual time scales than the subpolar MHT. Indeed, the RAPID MHT monthly standard deviation (0.28 PW) is about five times larger than that at OSNAP (0.051 PW).

Combining the OSNAP and RAPID MHT estimates reveals a convergence of heat transport in the NAD of 0.67 ± 0.24 PW during the overlapping time period of 2014–2018. The temporal variability of this heat transport convergence is largely dominated by the RAPID heat transport variability in the subtropics (see Fig. 4). The same situation probably holds before 2014, up to 2005, because the decadal changes in the subpolar heat transports are known to be weak during the past few decades (around a few tenth of PW, Li et al. 2021).

To extend our ocean heat budget analysis over the whole period of 2005–2018, we make the hypothesis that the mean MHT at OSNAP has not changed compared to August 2014 to May 2018 and is 0.50 ± 0.1 PW over 2005–2018. We also neglect the variability in OSNAP MHT before 2014 under the assumption that it has remained weak over the past decades and it plays only a marginal role (in comparison to the RAPID MHT variability) on the convergence of the heat transport over the NAD.

**Fig. 5:**
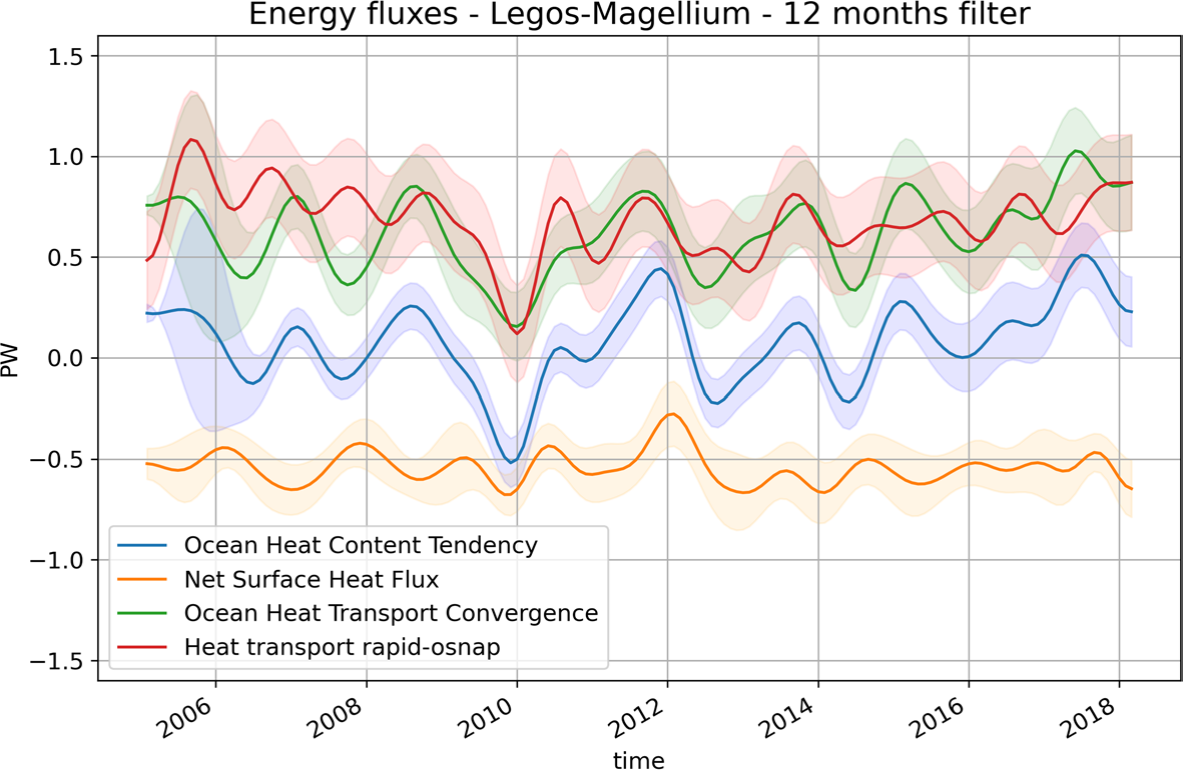
Monthly time series filtered with a Lanczos low-pass with a cutoff period at 1 year of the net surface heat flux (orange line) and OHCT estimated from Magellium/LEGOS (blue line) over the NAD (see Fig. 6 for OHCT time series derived from other OHCT products). Monthly time series series low-pass filtered at 1 year of the convergence of heat transport between the RAPID and the OSNAP sections estimated from in situ measurement of the RAPID array and the OSNAP array (red line) and from the residual of the oceanic energy budget (green line)

**Fig. 6:**
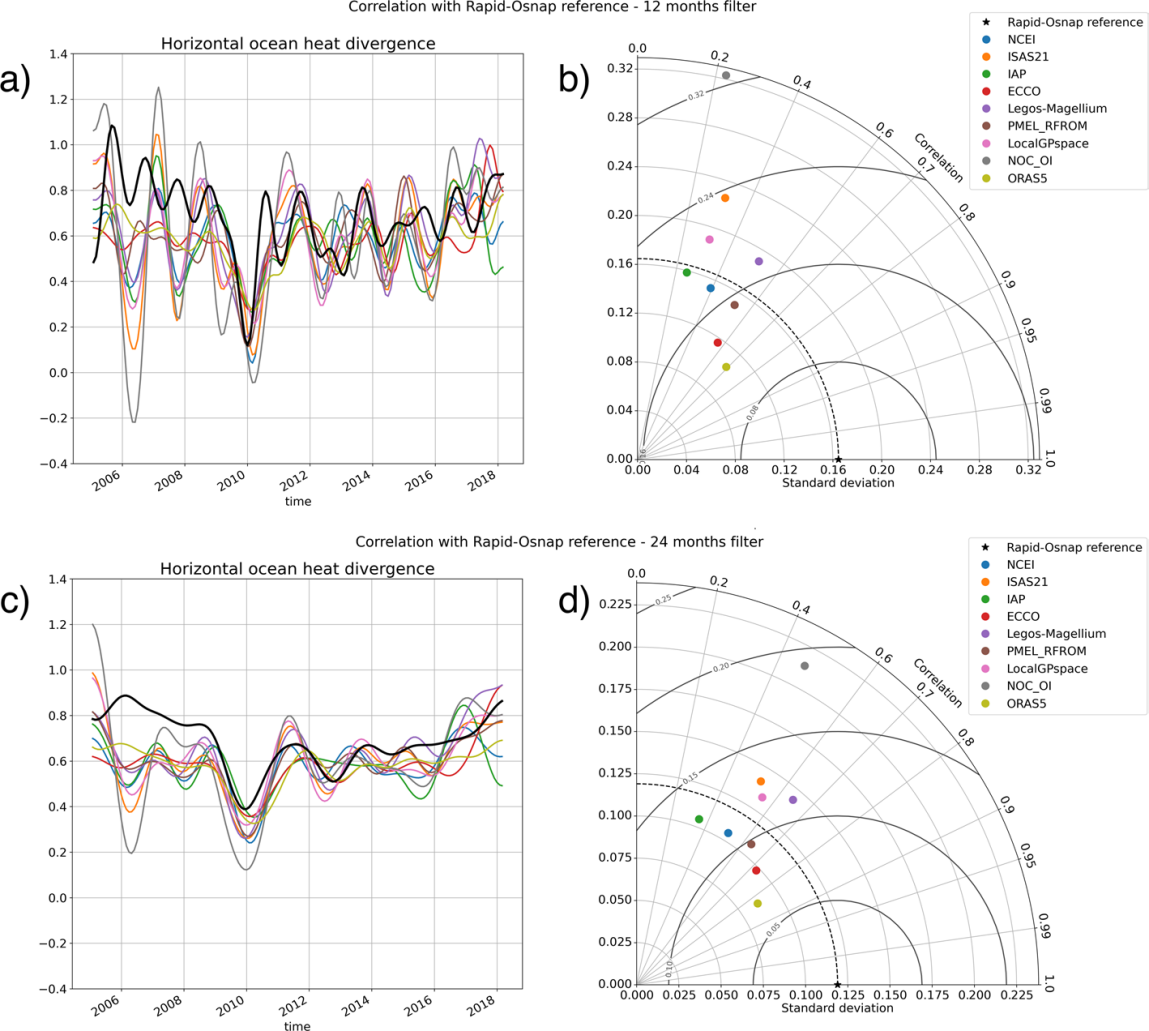
**a** Monthly time series low-pass filtered at 1 year of the meridional heat transport convergence between the RAPID and the OSNAP arrays estimated from in situ observations of the RAPID and the OSNAP arrays (black curve) and from the NAD energy budget residual with the OHCT derived from Magellium/LEGOS (purple line, same time series as the blue line in Fig. 5), RFROM (brown), NCEI (blue) ISAS21 (orange), IAP (green), ECCO (red), NOC_OI (gray), and LocalGPspace (pink). **b** Taylor diagram of the ocean heat transport convergence inferred from NAD energy budget residuals against the in situ estimate from the RAPID and the OSNAP arrays. **c** and **d** are, respectively, equivalent to **a** and **b** but with monthly time series low-pass filtered at 2 years

We estimate the total horizontal heat transport convergence in the NAD as the residual of the ocean energy budget spatially integrated between the RAPID section and the OSNAP section (Fig. 5, red line). We compare it with the estimate of the convergence of heat transport in the NAD estimated as the difference in MHT between the RAPID and OSNAP sections with RAPID and OSNAP in situ observations (red line). The components of the ocean energy budget, spatially integrated over the NAD, are also shown, including the OHCT (blue line) and the surface heat flux (orange line). Agreement between the heat transport convergence derived from the ocean energy budget and the in situ observations is reasonable both in terms of temporal mean and interannual variability. The difference in long-term means is 0.06 ± 0.4 PW, which is small (less than 15% of the signal) and not distinguishable from 0 given the level of uncertainty. The variability over 2008–2018 agrees well between both estimates with a correlation of 0.73 for annual time series (0.91 for biannual timeseries). Before 2008, the agreement in terms of variability is not as good. In addition, the in situ data show a decrease in heat transport convergence between 2005 and 2009 that is not captured by the ocean energy budget estimate. The decrease of heat transport convergence in 2009–2010, primarily wind-driven (McCarthy et al. 2012), lasts longer in the energy budget, until 2011. Over the whole record, the correlation between the in situ estimate and the energy budget estimate of the heat transport convergence is 0.54 and remains significant at the 95% confidence level (the confidence level is estimated with a two-tailed *p*-value test). On 2 year and longer time scales, agreement is better between both estimates of the NAD heat transport convergence with a correlation of 0.67 over the period of 2005–2018 (Fig. 6).

The energy budget estimate of the NAD allows us to analyze the causes of the temporal variability in the NAD OHCT and evaluate the role of heat transport convergence in NAD OHCT. We find that, on average over 2005–2018, the heat transport convergence in NAD is almost compensated by the NAD surface heat loss to the atmosphere leading to a 2005–2018 mean NAD OHCT of 0.062 ± 0.17 PW which is close to zero. On interannual time scales, the picture is different. The role of surface heat fluxes is sizable on the OHCT interannual variability but remains small except for some periods such as at the end of 2009 and at the beginning of 2012. The NAD heat convergence shows an increase from 2014 to 2018 that is captured by both the in situ estimate and the ocean energy budget. This increase results in a warming of the ocean in the NAD (OHCT positive) that is concomitant with a decrease of heat loss from the ocean to the atmosphere (decrease in surface heat flux).

All conclusions presented above hold for estimates of OHCT derived from any OHC product considered in this study. But, they show different levels of correlation, variance, and rms differences. Part of these differences in terms of correlation variance and rms differences is likely explained by the limited depth of several OHC products (indeed products based on in situ data except ISAS21, only cover the upper ocean down to 2000 m depth and probably miss important parts of the deeper branch of the AMOC) and their limited resolution. On annual time scales, products that use satellite altimetry (Magellium/LEGOS, RFROM, ECCO4) show better correlation between the energy budget estimate and the in situ estimate of the NAD heat transport convergence (Fig. 6a, b). They also show better or equivalent rms difference, but RFROM standard deviations are lower than that of the NAD heat transport convergence and the Magellium/LEGOS are higher (as are the NOC_OI standard deviations). On 2 year and longer time scales, the correlation increases for all product and the difference in correlation among them is reduced (Fig. 6a). For that time scale, the Magellium/LEGOS and NOC_OI standard deviations are still noticeably higher than the others. While the lateral heat transport convergence is cast as the reference in the Taylor diagrams, all of the terms in the heat budget have uncertainties and some errors can also be hidden in the estimate of the lateral heat transport convergence.

Generally, on 2 year and longer time scales, the energy budget estimates of the NAD heat transport convergence show similar temporal variability regardless of the product used for OHCT (Fig. 6b). Despite this similar temporal variability across energy budget estimates, some systematic differences remain with the in situ estimate of the NAD heat transport convergence, in particular before 2009 (see Fig. 6). These discrepancies could be due to some systematic errors common to all OHC content products. But, some of the OHC products (e.g., Magellium/LEGOS) are based on largely independent data. It rather suggests some errors in other terms of the energy budget such as the surface heat flux component, the assumption that the OSNAP MHT has not changed over 2005–2014, or the approximation associated with the net zero mass transport constraint applied to RAPID and OSNAP heat transport calculation. The latter option is unlikely given the magnitude of the difference between the energy-based and the in situ-based estimate of the NAD heat transport convergence, in particular before 2009. Previous studies also suspected some issue in the in situ estimate of the RAPID MHT before 2009 (Trenberth and Fasullo 2017).

On annual time scales, the situation is slightly different as we find some significant differences in the temporal variability across the energy budget-based estimates of the NAD heat transport convergence. The differences are particularly large during 2009 and 2011–2014. They may be due to several issues including mapping errors, differences in the climatology, differences in the data quality criteria, and others.

## Discussion and Conclusions

In this study, we evaluate the heat transport convergence over the North Atlantic with an energy budget approach using different ocean heat content products derived from in situ temperature measurements, satellite altimetry, satellite gravimetry data, and reanalysis. We compare the energy-based estimates of the North Atlantic heat transport convergence with an estimate derived from the RAPID and OSNAP arrays’ in situ data over the period of 2005–2018. Confirming previous studies (Trenberth and Fasullo 2017, Forget and Ferreira 2019, Liu et al. 2020, Mayer et al. 2022), we find a good agreement in terms of mean (agreement at precision of ± 0.24 PW) and a reasonable agreement in terms of annual and interannual temporal variability between the energy-based estimates and the in situ-based estimate of the North Atlantic heat transport convergence (with significant correlations between 0.22 and 0.7 for time series low-pass filtered at 1 year). The agreement in temporal variability increases at 2-year and longer time scales (with significant correlations between 0.35 and 0.83 for time series low-pass filtered at 2 years). We close the oceanic energy budget in the North Atlantic Ocean to within ± 0.2 PW (residual STD) for the closed ocean domain between RAPID array and the OSNAP array with an average budget residual of 0.06 PW (residual mean).

We find that the temporal mean spatial variability of the local North Atlantic heat transport convergence is compensated for by the local time-mean surface heat fluxes leading to a time-mean OHCT that is closed to uniform over 2005–2018 in the NAD. However, it is the North Atlantic heat transport convergence that explains most of the time variability of OHCT averaged over the NAD. So, the agreement in terms of annual and interannual temporal variability between the energy-based and the in situ-based estimate of the North Atlantic heat transport convergence means that the OHC products contain part of the information associated to the MHT. This is true at 2 year and longer time scales for all OHC products tested but less so at annual time scales where OHC products using satellite altimetry tend to perform better. The comparisons made here provide an independent framework for assessing regional ocean heat content and their capacity to represent the ocean heat transport means and variability, in particular, the heat transport associated with the main branches of the overturning circulation (Forget and Ferreira 2019; Rousselet et al 2021). Such validation is useful if ocean heat content products are to be used in objective regionalized Earth energy budget to evaluate the changes in regional energy fluxes as in the GEWEX effort from Stephens et al. (2023). In this respect, continuous monitoring of the heat transport at ocean cross-sections such as RAPID and OSNAP is essential to test the closure of ocean energy budget and validate ocean heat content estimates at regional scale.

The OHC product that leads to the highest correlation between the energy-based and the in situ-based estimates of the NAD heat transport convergence on annual time scales is based on satellite altimetry and space gravimetry data. It shows a correlation of 0.54 for annual time series over 2005–2018 (0.67 for biannual time series). This is comparable to Trenberth and Fasullo (2017) but smaller than Liu et al. (2020) who obtained a correlation of 0.66 and Mayer et al. (2022) who obtained a correlation of 0.72. However, our approach is different from Trenberth and Fasullo (2017) and Liu et al. (2020) attempts, which inferred OHT between the RAPID section and the Bering Strait, and from Mayer et al. (2022) attempt which inferred OHT between the RAPID section and the Greenland–Scotland Ridge and Davis Strait. It is unlikely though that the difference in the domain size explain the difference in correlation as the time variability in heat transport convergence over the North Atlantic is generally dominated by the RAPID MHT variability. Liu et al. (2020) and Mayer et al (2022) have in common that they use the ocean reanalysis ORAS5 to infer the OHCT. We tested ORAS5 to derive the OHCT, and we found indeed a better correlation of 0.7 (for annual time series and 0.82 for time series filtered at 2 years). A simple interpretation of this better performance of ORAS5 is that ORAS5 better resolves the Atlantic MHT in its OHC reconstruction. However, ORAS5 is an ocean reanalysis which uses ERA-interim atmospheric reanalysis for the surface forcing. Over the common period 2005–2014, ERA-interim surface heat fluxes averaged over the NAD are close to ERA5 surface fluxes (not shown). We suspect that using a similar surface heat flux to force ORAS5 reanalysis and to infer the ocean energy budget, leads to compensation of errors in Liu et al. (2020) and Mayer et al (2022). In our study, these errors cannot compensate and may lead to a poorer correlation with in situ-based estimate of the heat transport convergence. More analyses are needed to test this hypothesis.

The drop from 2005 to 2009 in NAD heat transport convergence observed by the in situ data of RAPID and OSNAP is not captured by any energy budget estimate of this study, regardless of the OHC product used. It is not captured by previous energy budget studies either (Trenberth and Fasullo 2017, Liu et al. 2020, Mayer et al. 2022). In this study, we find that this discrepancy is unlikely related to the OHC data as we tested different estimates of OHC based on independent data. It raises questions on the cause for this discrepancy. Is it an issue in surface heat fluxes? Or in the in situ data? How substantial is the vertical heat flux through the 2000 m (the bottom depth of most of the ocean heat content estimates) in the NAD? Can we really assume no changes in the MHT variability across the OSNAP section before 2014?

Although we find the North Atlantic Ocean energy budget is closed on annual time scales, there is room for improvement. It would be interesting to get to monthly estimates but this objective remains challenging for the argo observing system and for the space gravimetry observing system, but also the in situ-measurements in the ocean. In addition, the processing of the convergence (interpolation and truncation) and the processing of the tendency (derivation) both amplify the noise and introduce uncertainties that are larger for higher temporal and spatial resolution. These issues pose a challenge as well.

## Data Availability

The authors have no financial or proprietary interests in any material discussed in this article.

## References

[CR1] Blazquez A, Meyssignac B, Lemoine J, Berthier E, Ribes A, Cazenave A (2018) Exploring the uncertainty in GRACE estimates of the mass redistributions at the Earth surface: implications for the global water and sea level budgets. Geophys J Int 215:415–430. 10.1093/gji/ggy293

[CR2] Bryden HL, Imawaki S (2001) Ocean heat transport. Int Geophys. Acad Press 77:455–474

[CR4] Cheng L, Zhu J (2016) Benefits of CMIP5 multimodel ensemble in reconstructing historical ocean subsurface temperature variation. J Climate 29:5393–5416. 10.1175/JCLI-D-15-0730.1

[CR5] Cheng L, Trenberth KE, Fasullo J, Boyer T, Abraham J, Zhu J (2017) Improved estimates of ocean heat content from 1960–2015. Sci Adv 3:e1601545. 10.1126/sciadv.1601545 28345033 10.1126/sciadv.1601545 PMC5345929

[CR6] Collins M, Sutherland M, Bouwer L, Cheong S-M, Frölicher, Jacot Des Combes H, Koll Roxy M, Losada I, McInnes K, Ratter B, Rivera-Arriaga E, Susanto RD, Swingedouw D, Tibig L (2019) Extremes, abrupt changes and managing risk. In: IPCC special report on the ocean and cryosphere in a changing climate [Pörtner H-O, Roberts DC, Masson-Delmotte V, Zhai P, Tignor M, Poloczanska E, Mintenbeck K, Alegría A, Nicolai M, Okem A, Petzold J, Rama B, Weyer NM (eds)]. In press.

[CR7] Dee DP et al (2011) The ERA-Interim reanalysis: configuration and performance of the data assimilation system. Q J R Meteorol Soc 137:553–597

[CR8] Forget G, Ferreira D (2019) Global ocean heat transport dominated by heat export from the tropical Pacific. Nat Geosci 12:351–354. 10.1038/s41561-019-0333-7

[CR9] Forget G, Ponte RM (2015) The partition of regional sea level variability. Prog Oceanogr 137:173–195

[CR10] Forget G, Campin J-M, Heimbach P, Hill CN, Ponte RM, Wunsch C (2015a) ECCO version 4: an integrated framework for non-linear inverse modeling and global ocean state estimation. Geosci Model Dev 8:3071–3104. 10.5194/gmd-8-3071-2015

[CR11] Forget G, Ferreira D, Liang X (2015b) On the observability of turbulent transport rates by Argo: supporting evidence from an inversion experiment. Ocean Sci 11(5):839–853

[CR12] Frajka-Williams E et al (2019) Atlantic meridional overturning circulation: observed transport and variability. Front Mar Sci 6:260. 10.3389/fmars.2019.00260

[CR13] Fukumori I, Wang O, Fenty I, Forget G, Heimbach P, Ponte RM (2021) Synopsis of the ECCO central production global ocean and sea-ice state estimate, version 4 release 4 (4 release 4). Zenodo. 10.5281/zenodo.4533349

[CR15] Gaillard F, Reynaud T, Thierry V, Kolodziejczyk N, von Schuckmann K (2016) In situ-based reanalysis of the global ocean temperature and salinity with ISAS: variability of the heat content and steric height. J Clim 29:1305–1323. 10.1175/JCLI-D-15-0028.1

[CR16] Ganachaud A, Wunsch C (2000) Improved estimates of global ocean circulation, heat transport and mixing from hydrographic data. Nature 408:453–457 11100723 10.1038/35044048

[CR17] Giglio D., Sukianto T., Kuusela M. (2023). Ocean heat content anomalies in the North Atlantic based on mapping Argo data using local Gaussian processes defined over space (1.0.0). enodo. 10.5281/zenodo.10183869

[CR19] Hersbach H, Bell B, Berrisford P, Hirahara S, Horányi A, Muñoz-Sabater J, Thépaut JN (2020) The ERA5 global reanalysis. Quart J Roy Meteor Soc 146:1999–2049. 10.1002/qj.3803

[CR20] Jackson LC, Dubois C, Forget G, Haines K, Harrison M, Iovino D et al (2019) The mean state and variability of the North Atlantic circulation: A perspective from ocean reanalyses. J Geophys Res: Oceans 124:9141–9170. 10.1029/2019JC015210

[CR21] Johns WE et al (2011) Continuous, array-based estimates of Atlantic Ocean heat transport at 26.5°N. J Clim 24:2429–2449

[CR22] King BA (2023) Objectively mapped Argo profiling float data and RAPID moored microcat data from the North Atlantic Ocean, 2004–2022. NERC EDS Br Oceanogr Data Centre NOC. 10.5285/fe8e524d-7f04-41f3-e053-6c86abc04d51

[CR24] Kolodziejczyk N, Prigent-Mazella A, Gaillard F (2021). ISAS temperature and salinity gridded fields. SEANOE. 10.17882/52367

[CR25] Kolodziejczyk Nicolas, Prigent-Mazella Annaig, Gaillard Fabienne (2023). ISAS temperature, salinity, dissolved oxygen gridded fields. SEANOE. 10.17882/52367

[CR26] Kuusela M., Stein M.L. (2018) Locally stationary spatio-temporal interpolation of Argo profiling float data. In: Proc. R. Soc. A, 474, pp 20180400. 10.1098/rspa.2018.0400 10.1098/rspa.2018.0400 PMC6304028 30602929

[CR27] L’Ecuyer TS, Beaudoing HK, Rodell M, Olson W, Lin B, Kato S, Clayson CA et al (2015) The observed state of the energy budget in the early twenty-first century. J Clim 21:8319–8346

[CR28] Legeais J-F, Meyssignac B, Faugère Y, Guerou A, Ablain M, Pujol M-I, Dufau C, Dibarboure G (2021) Copernicus sea level space observations: a basis for assessing mitigation and developing adaptation strategies to sea level rise. Front Mar Sci. 10.3389/fmars.2021.704721

[CR29] Levitus S, Antonov JI, Boyer TP, Locarnini RA, Garcia HE, Mishonov AV (2009) Global ocean heat content 1955–2008 in light of recently revealed instrumentation problems. Geophys Res Lett. 10.1029/2008GL037155

[CR30] Levitus S et al (2012) World ocean heat content and thermosteric sea level change (0–2000 m), 1955–2010. Geophys Res Lett 39:L10603. 10.1029/2012GL051106

[CR31] Li F, Lozier M, Holliday N, Johns W, Le Bras I, Moat B, Cunningham S, de Jong M (2021) Observation-based estimates of heat and freshwater exchanges from the subtropical North Atlantic to the Arctic. Prog Oceanogr 197:102640

[CR90] Liu C et al (2020) Variability in the global energy budget and transports 1985-2017. Climate Dyn 55:3381–3396. 10.1007/s00382-020-05451-8

[CR99] Locarnini RA, Mishonov AV, Antonov JI, Boyer TP, Garcia HE (2010) World Ocean Atlas 2009, Volume1: Temperature. In: S. Levitus, Ed., NOAA Atlas NESDIS 68, U.S. Gov. Printing Office, Washington, D.C., pp 184

[CR33] Loeb NG, Thorsen TJ, Norris JR, Hailan W, Wenying S (2018) Changes in Earth’s energy budget during and after the “pause” in global warming: an observational perspective. Climate 6(3):62. 10.3390/cli6030062

[CR34] Lozier MS, Li F, Bacon S, Bahr F, Bower AS, Cunningham SA, de Jong MF, de Steur L, de Young B, Fischer J, Gary SF, Greenan BJW, Holliday NP, Houk A, Houpert L, Inall ME, Johns WE, Johnson HL, Johnson C, Karstensen J, Koman G, Le Bras IA, Lin X, Mackay N, Marshall DP, Mercier H, Oltmanns M, Pickart RS, Ramsey AL, Rayner D, Straneo VF, Thierry Torres DJ, Williams RG, Wilson C, Yang J, Yashayaev I, Zhao I (2019) A sea change in our view of overturning in the subpolar North Atlantic. Science 363(6426):516–521 30705189 10.1126/science.aau6592

[CR35] Lyman JM, Johnson GC (2023) Global high-resolution random forest regression maps of ocean heat content anomalies using in situ and satellite data. J Atmos Ocean Technol 40(5):575–586. 10.1175/JTECH-D-22-0058.1

[CR36] Macdonald AM, Candela J, Bryden HL (1994) An estimate of the net heat transport through the Strait of Gibraltar. In: Seasonal and Interannual Variability of the Western Mediterranean Sea, LaViolette PE, (ed) Amer Geophys. Union, pp 13–32

[CR37] Magellium/LEGOS: Atlantic OHC from space: Heat content change over the Atlantic Ocean by space geodetic approach, 10.24400/527896/A01-2022.012, 2022.

[CR39] Marti F, Blazquez A, Meyssignac B, Ablain M, Barnoud A, Fraudeau R, Jugier R, Chenal J, Larnicol G, Pfeffer J, Restano M, Benveniste J (2022) Monitoring the ocean heat content change and the Earth energy imbalance from space altimetry and space gravimetry. Earth Syst Sci Data 14:229–249. 10.5194/essd-14-229-2022

[CR95] Marti F, Meyssignac B, Rousseau V, Ablain M, Fraudeau R, Blazquez A, Fourest S (2024) Monitoring global ocean heat content from space geodetic observations to estimate the Earth energy imbalance. In: von Schuckmann K, Moreira L, Grégoire M, Marcos M, Staneva J, Brasseur P, Garric G, Lionello P, Karstensen J, Neukermans G (eds) 8th edition of the Copernicus Ocean State Report (OSR8). Copernicus Publications, State Planet, 4-osr8, 3. 10.5194/sp-4-osr8-3-2024

[CR41] Mayer J, Haimberger MML (2021) Consistency and homogeneity of atmospheric energy, moisture, and mass budgets in ERA5. J Climate 34:3955–3974. 10.1175/JCLI-D-20-0676.1

[CR100] Mayer M, Haimberger L, Edwards JM, Hyder P (2017) Toward consistent diagnostics of the coupled atmosphere and ocean energy budgets. J Climate 30:9225–9246. 10.1175/JCLI-D-17-0137.1

[CR42] Mayer J, Mayer M, Haimberger L (2021) Mass-consistent atmospheric energy and moisture budget monthly data from 1979 to present derived from ERA5 reanalysis. Copernic Clim Change Serv (C3S) Clim Data Store (CDS). 10.24381/cds.c2451f6b

[CR43] Mayer J, Mayer M, Haimberger L, Liu C (2022) Comparison of surface energy fluxes from global to local scale. J Clim. 10.1175/JCLI-D-21-0598.1

[CR96] Mayer M, Kato S, Bosilovich M et al (2024) Assessment of atmospheric and surface energy budgets using observation-based data products. Surv Geophys. https://doi-org.insu.bib.cnrs.fr/10.1007/s10712-024-09827-x 10.1007/s10712-024-09827-x PMC11671425 39734430

[CR44] McCarthy G, Frajka-Williams E, Johns WE, Baringer MO, Meinen CS, Bryden HL, Rayner D, Duchez A, Roberts CD, Cunningham SA (2012) Observed interannual variability of the Atlantic meridional overturning circulation at 26.5°N. Geophys Res Lett 39:L19609. 10.1029/2012GL052933

[CR45] McCarthy GD, Smeed DA, Johns WE, Frajka-Williams E, Moat BI, Rayner D, Baringer MO, Meinen CS, Collins J, Bryden HL (2015) Measuring the Atlantic meridional overturning circulation at 26^°^N. Prog Oceanogr 130:91–111

[CR46] Mecking JV, Drijfhout SS (2023) The decrease in ocean heat transport in response to global warming. Nat Clim Change. 10.1038/s41558-023-01829-8 10.1038/s41558-023-01829-8 PMC13538074 42694091

[CR47] Melet A, Meyssignac B (2015) Explaining the spread in global mean thermosteric sea level rise in CMIP5 climate models. J Clim 28:9918–9940. 10.1175/JCLI-D-15-0200.1

[CR48] Palter JB (2015) The role of the Gulf stream in European climate. Ann Rev Mar Sci 7(1):113–137 10.1146/annurev-marine-010814-015656 25560606

[CR49] Prandi P, Meyssignac B, Ablain M, Spada G, Ribes A, Benveniste J (2021) Local sea level trends, accelerations and uncertainties over 1993–2019. Sci Data 8:1. 10.1038/s41597-020-00786-7 33414438 10.1038/s41597-020-00786-7 PMC7791125

[CR50] Rhines P, Häkkinen S, Josey SA (2008) Is oceanic heat transport significant in the climate system? In: Dickson RR, Meincke J, Rhines P (eds) Arctic-Subarctic Ocean Fluxes. Springer, Dordrecht

[CR52] Rousselet L, Cessi P, Forget G (2021) Coupling of the mid-depth and abyssal components of the global overturning circulation according to a state estimate. Sci Adv 7:eabf5478. 10.1126/sciadv.abf5478 34020953 10.1126/sciadv.abf5478 PMC11792135

[CR54] Stephens G, Polcher J, Zeng X, Van Oevelen P, Poveda G, Bosilovich M, Ahn MH, Balsamo G, Duan Q, Hegerl G, Jakob C (2023) The first 30 years of GEWEX. Bull Amer Meteor Soc 104:E126–E157. 10.1175/BAMS-D-22-0061.1

[CR55] Storto A et al (2019) Ocean reanalyses: recent advances and unsolved challenges. Front Mar Sci 6:418

[CR56] Trenberth KE, Caron JM (2001) Estimates of meridional atmosphere and ocean heat transports. J Clim 14:3433–3443

[CR58] Trenberth KE, Fasullo JT (2017) Atlantic meridional heat transports computed from balancing Earth’s energy locally. Geophys Res Lett 44:1919–1927. 10.1002/2016GL072475

[CR59] Wu P, Haines K (1998) The general circulation of the Mediterranean Sea from a 100-year simulation. J Geophys Res 103:1121–1135. 10.1029/97JC02720

[CR60] Zuo H, Balmaseda MA, Tietsche S, Mogensen K, Mayer M (2019) The ECMWF operational ensemble reanalysis–analysis system for ocean and sea ice: a description of the system and assessment. Ocean Sci 15(3):779–808. 10.5194/os-15-779-2019

[CR61] Zuo H, Balmaseda MA, de Boisseson E, Tietsche S, Mayer M, de Rosnay P, (2021) The ORAP6 ocean and sea-ice reanalysis: Description and evaluation. EGU General Assembly Conference Abstracts, EGU21–9997, EGU General Assembly Conference Abstracts.

